# *Andrographis paniculata* Inhibits Tongue Squamous Cell Carcinoma via Regulating Wnt/β-Catenin Signaling and Epithelial-Mesenchymal Transition

**DOI:** 10.3390/ijms27093772

**Published:** 2026-04-23

**Authors:** Grace Gar-Lee Yue, Jingyi Huang, Xiaotong Lu, Julia Kin-Ming Lee, Si Gao, Jason Ying Kuen Chan, Clara Bik-San Lau

**Affiliations:** 1 Department of Pharmacology and Pharmacy, Li Ka Shing Faculty of Medicine, The University of Hong Kong, Pokfulam, Hong Kong SAR, China; 2 Institute of Chinese Medicine, The Chinese University of Hong Kong, Shatin, Hong Kong SAR, China; 3 Department of Otorhinolaryngology, Head and Neck Surgery, The Chinese University of Hong Kong, Shatin, Hong Kong SAR, China; 4 School of Chinese Medicine, Li Ka Shing Faculty of Medicine, The University of Hong Kong, Pokfulam, Hong Kong SAR, China

**Keywords:** *Andrographis paniculata*, tongue squamous cell carcinoma, Wnt/β-catenin signaling, mitochondrial apoptosis, epithelial–mesenchymal transition

## Abstract

Tongue squamous cell carcinoma (TSCC) is an aggressive malignancy with poor prognosis and limited therapeutic options. Herbal medicines with multitarget activities and low toxicity have attracted increasing attention in cancer adjuvant therapy. This study aimed to investigate the anti-tumor effects and underlying mechanisms of the water extract of *Andrographis paniculata* (APW) in TSCC in vitro and in vivo. Two TSCC cell lines, Cal-27 and SCC25, were used for cell-based functional and mechanistic studies, while a Cal-27 xenograft-bearing mouse model was established for evaluating the in vivo effect of APW treatment. Our results showed that APW could significantly inhibit the proliferation of Cal-27 and SCC25 cells and induce apoptosis in a concentration-dependent manner. APW could promote mitochondrial-mediated apoptosis by upregulating Bax and cleaved caspase proteins but downregulating Bcl-2 in TSCC cells. It also suppressed the Wnt/β-catenin signaling pathway, reducing β-catenin expression and its downstream targets, CCND1, MYC, and JUN. Furthermore, APW disrupted mitochondrial integrity, induced cytochrome c release, and reduced mitochondrial membrane potential. APW also inhibited epithelial–mesenchymal transition, increasing E-cadherin and decreasing N-cadherin and vimentin expressions, thereby suppressing cell migration of TSCC cells. Furthermore, the 5-week APW treatment significantly reduced tumor growth and angiogenesis without evident hepatic or renal toxicity in Cal-27 xenograft-bearing mice. In conclusion, APW exerted potent anti-tumor effects by targeting both the Wnt/β-catenin pathway and mitochondrial apoptotic machinery, suggesting the great potential of APW as an adjuvant therapeutic candidate for TSCC treatment.

## 1. Introduction

Tongue squamous cell carcinoma (TSCC) is a subtype of oral squamous cell carcinoma and among the most aggressive malignancies in the head and neck region [1]. It is characterized by rapid local invasion, a high metastatic potential, and an overall poor prognosis [2,3]. Tobacco and alcohol consumption, along with human papillomavirus (HPV) infection, are well-established etiological factors [4,5]. Due to the rich lymphatic drainage of the tongue, TSCC frequently exhibits early local invasion and metastasis to cervical lymph nodes, which occur in approximately 40% of patients at initial diagnosis [6]. The treatments include surgery, radiotherapy, and chemotherapy, which are effective; however, the 5-year survival rate for advanced TSCC remains unsatisfactory, underscoring the need for new therapeutic agents with improved efficacy and reduced toxicity.

Herbal medicines and natural products have long been a valuable source of anticancer agents [7,8]. *Andrographis paniculata* (Burm.f.) Wall. ex Nees, also named Chuan Xin Lian in Chinese and Kalmegh in Indian, is widely used in China and Southeast Asia for its anti-inflammatory and anti-microbial properties. Its indications in the Chinese Pharmacopeia (2020 version) also include mouth and tongue sores. Our previous studies have shown the potent anti-metastatic activities of *Andrographis paniculata* (AP) water extract in esophageal squamous cell carcinoma (ESCC) by regulating key molecules involved in cell proliferation, migration, and metastasis [9,10,11]. Network pharmacology and experimental validation in esophageal cancer have identified several active compounds of AP, including panicolin and moslosooflavone, which target critical signaling molecules such as EGFR, AKT, and STAT3 to modulate cancer-related pathways [12]. The components of APW, such as andrographolide, bisandrographolide A, and bisandrographolide C, could bind to and suppress the function of tetraspanin CD81 in ESCC cells [13]. Based on the findings in ESCC, we hypothesized that TSCC would also be sensitive to APW treatment. Furthermore, the potential therapeutic effects of APW and underlying mechanisms in TSCC remain unexplored, warranting a systematic investigation.

The Wnt/β-catenin signaling pathway always plays a pivotal role in regulating proliferation and differentiation of cells, apoptosis, and epithelial–mesenchymal transition (EMT) during tumor progression [14,15,16]. Abnormal activation of the Wnt/β-catenin pathway has been widely implicated in tumor initiation, growth, invasion, metastasis, and resistance to therapy in various cancers, including oral squamous cell carcinoma [17]. Besides, mitochondrial-mediated apoptosis, characterized by an imbalance between pro-apoptotic protein Bax and anti-apoptotic protein Bcl-2, release of cytochrome c, and loss of mitochondrial membrane potential, constitutes a classical intrinsic death pathway and represents a critical mechanism through which anticancer agents exert their effects [18,19]. Notably, accumulating evidence indicates a functional relationship between Wnt/β-catenin signaling and mitochondrial integrity, whereby modulation of one pathway can influence the other, potentially amplifying the overall apoptotic response in cancer cells [20,21,22]. In view of this, we hypothesized that APW could induce apoptosis in TSCC cells through coordinated suppression of Wnt/β-catenin signaling and mitochondrial dysfunction.

Hence, the present study aimed to explore the potential anti-tumor effects of APW on TSCC both in vitro and in vivo. It also aimed to verify whether APW suppresses tumor growth and metastasis by inhibiting the Wnt/β-catenin pathway, promoting mitochondrial-mediated apoptosis, and suppressing EMT.

## 2. Results

### 2.1. APW Inhibited TSCC Cells Proliferation

**Figure 1:**
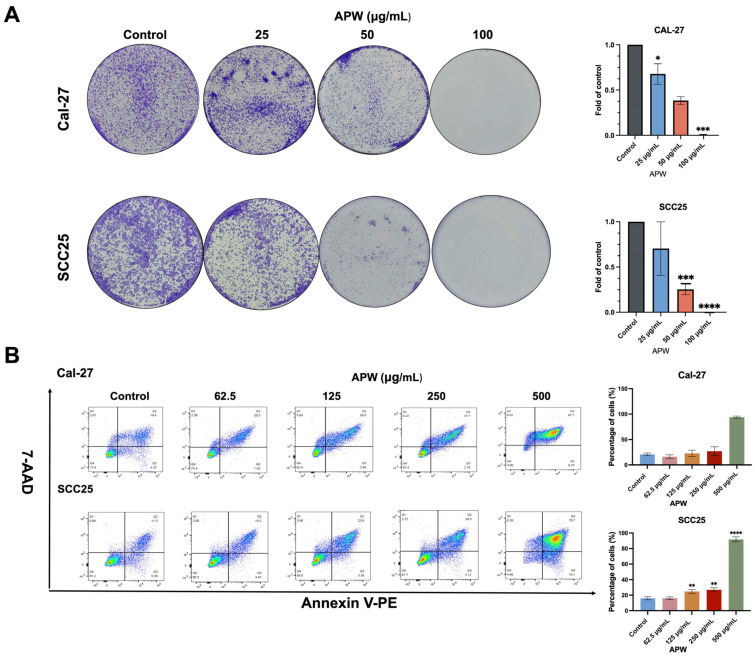
APW inhibited colony formation and induced apoptosis in Cal-27 and SCC25 cells. (**A**) Cal-27 and SCC25 cells were treated with increasing concentrations of APW (25, 50, 100 μg/mL) for 48 h. Representative images of colonies are shown on the left panel, and quantification of colony numbers relative to control is shown on the right panel. (**B**) Cal-27 and SCC25 cells treated with APW (62.5, 125, 250, 500 μg/mL) for 48 h were subjected to flow cytometry analysis of apoptosis using Annexin V-PE/7-AAD staining. Representative dot plots are shown on the left panel, and the percentage of apoptotic cells (early + late apoptosis) is quantified on the right panel. Data are presented as mean ± SD (*n* = 3); * *p* < 0.05, ** *p* < 0.01, *** *p* < 0.001, and **** *p* < 0.0001 versus control.

The cell viability of TSCC cells after APW treatment was assessed using an MTT assay. The IC_50_ values of APW in Cal-27 and SCC25 were 128.6 and 309.9 µg/mL, respectively. While the IC_50_ values of cisplatin in Cal-27 and SCC25 were 12.1 and 15.2 µM, respectively. As shown in Figure 1A, APW significantly inhibited the proliferation of Cal-27 and SCC25 cells. Notably, when the APW concentration was raised to 500 µg/mL, nearly 80% of cells underwent apoptosis (Figure 1B).

### 2.2. APW Induced Apoptosis in TSCC Cells

**Figure 2:**
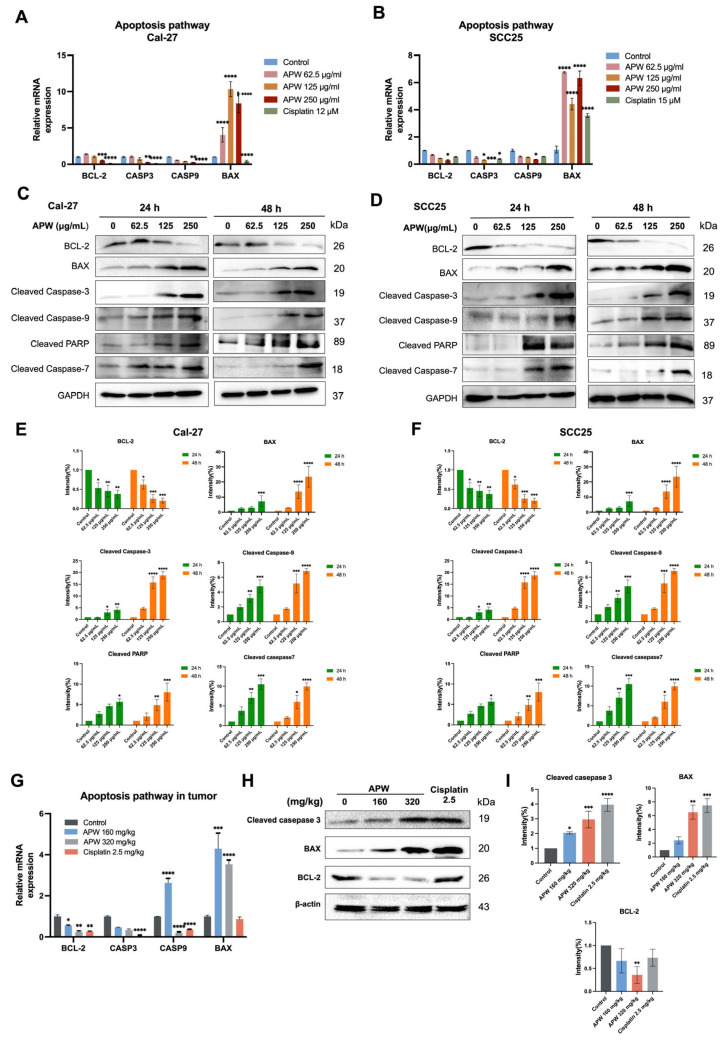
APW induced apoptosis in Cal-27 and SCC25 cells through activation of the caspase-dependent pathway. (**A**) Cal-27 and (**B**) SCC25 cells treated with various concentrations of APW (62.5, 125, and 250 μg/mL) for 24 h and subjected to RT–qPCR analysis of apoptosis-related genes (BCL-2, CASP3, CASP9, and BAX). Cisplatin (12 μM in Cal-27 and 15 μM in SCC25 cells) was used as a positive control. (**C**) Cal-27 and (**D**) SCC25 cells treated with APW (62.5, 125, and 250 μg/mL) for 24 h or 48 h and then subjected to Western blot analysis of apoptosis-associated proteins. (**C**,**D**) Representative blots of proteins and (**E**,**F**) quantification of protein band intensities were shown. Data are expressed as mean ± SD (*n* = 3). Tumor tissues of mice treated with APW (160 or 320 mg/kg) or cisplatin (2.5 mg/kg) were collected for RNA and protein extraction. (**G**) Relative mRNA expressions of apoptosis-related genes (BCL-2, CASP3, CASP9, and BAX) in tumor tissues was determined using RT-qPCR. (**H**,**I**) Protein expression of cleaved caspase-3, BAX, and BCL-2 in tumor tissues were examined using Western blot analysis. (**H**) Representative blots of proteins and (**I**) quantification of protein band intensities were shown. Data were presented as mean ± SEM, *n* = 12–14 in each group, and statistical significance was determined using one-way ANOVA, with * *p* < 0.05, ** *p* < 0.01, *** *p* < 0.001, and **** *p* < 0.0001 versus control.

To elucidate the molecular mechanisms underlying APW-induced apoptosis in TSCC cells, the expressions of apoptosis-related genes and proteins in Cal-27 and SCC25 cells, as well as in xenograft tumor tissues, were examined. RT-qPCR data analysis showed that APW treatment significantly up-regulated expression of the pro-apoptotic gene BAX, while the anti-apoptotic gene BCL-2 was markedly down-regulated in both cell lines (Figure 2A,B). In addition, several key components of the caspase cascade exhibited transcriptional suppression after APW treatment, suggesting post-transcriptional regulation. Consistently, analysis of tumor tissues from APW-treated mice demonstrated a similar pattern of increased BAX and decreased BCL-2 expressions (Figure 2G).

At protein levels of TSCC cells, western blot results corroborated the qPCR data that APW treatment led to a robust upregulation of BAX and a significant reduction in BCL-2 protein expressions. Furthermore, treatment with APW further activated the apoptotic cascade, reflected by a concentration-dependent accumulation of cleaved apoptotic caspase-3 and -9 (Figure 2C–F). In contrast, the levels of the uncleaved caspases-3, -7, and -9 and PARP showed either no marked change or a reduction in both Cal-27 and SCC25 cells (Supplementary Information Figure S2). Similar results were observed in tumor tissues, where APW treatment resulted in up-regulation of the expression of cleaved apoptotic proteins (Figure 2H,I).

Together, these data demonstrate that APW promotes apoptosis in tongue cancer cells by modulating the BCL-2/BAX balance and activating the caspase cascade.

### 2.3. APW Inhibited Wnt/β-Catenin Signaling Pathway in TSCC Cells

**Figure 3:**
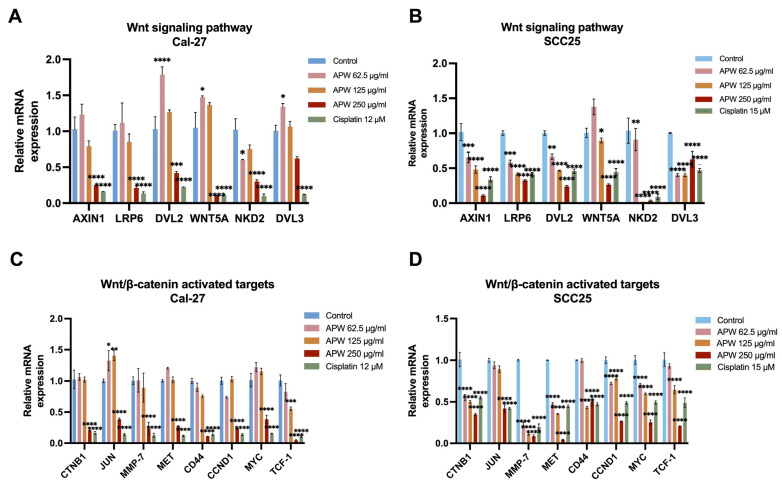
APW down-regulated the Wnt/β-catenin signaling pathway in Cal-27 and SCC25 cells. Cal-27 and SCC25 cells were treated with APW (62.5, 125, or 250 μg/mL) or cisplatin (12 μM in Cal-27 cells or 15 μM in SCC25 cells) for 48 h and subjected to RT–qPCR analysis. Expressions of (**A**,**B**) Wnt signaling components (AXIN1, LRP6, DVL2, WNT5A, NKD2, and DVL3) and (**C**,**D**) downstream target genes (CTNB1, JUN, MMP-7, MET, CD44, CCND1, MYC, and TCF1) were normalized to GAPDH and presented as mean ± SD (*n* = 3). Statistical significance was determined using one-way ANOVA, with * *p* < 0.05, ** *p* < 0.01, *** *p* < 0.001, and **** *p* < 0.0001 versus control.

The Wnt/β-catenin signaling pathway was suggested to play crucial roles in regulating apoptosis and tumor progression [23,24]. Since APW was shown to markedly induce apoptosis in TSCC cells, the involvement of the Wnt pathway was further investigated. The RT-qPCR results showed that APW treatment significantly decreased the mRNA levels of several key Wnt pathway components, including LRP6, DVL2, DVL3, NKD2, and WNT5A, in a dose-dependent manner in both Cal-27 and SCC25 cell lines (Figure 3A,B). Moreover, the mRNA expressions of β-catenin–activated target genes such as CTNNB1, JUN, MYC, and CCND1 were also down-regulated (Figure 3C,D).

**Figure 4:**
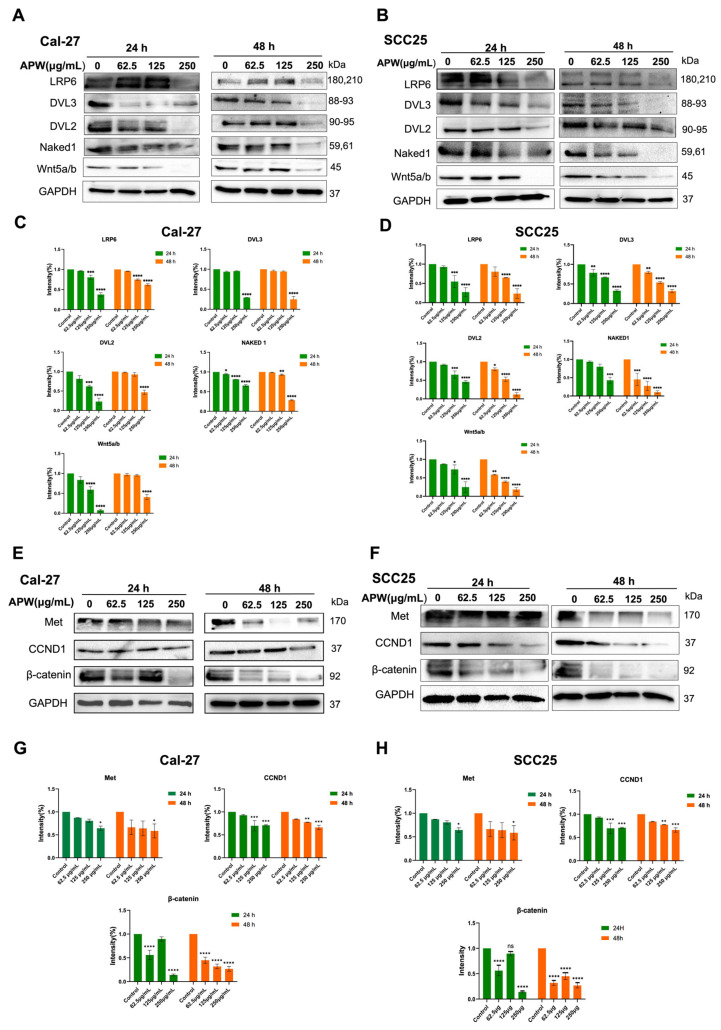
APW suppressed the expressions of proteins in Wnt/β-catenin signaling pathway in Cal-27 and SCC25 cells. Cal-27 and SCC25 cells were treated with APW (62.5, 125, 250 μg/mL) for 24 h or 48 h and subjected to Western blot analysis. Expressions of Wnt pathway components (LRP6, DVL3, DVL2, Naked1, and Wnt5a) and downstream targets (Met, CCND1, and β-catenin) was shown in representative blots of (**A**,**E**) Cal-27 cells and (**B**,**F**) SCC25 cells. Quantification of band intensities were shown in (**C**,**D**,**G**,**H**). Data are expressed as mean ± SD (*n* = 3). Statistical significance was determined using one-way ANOVA, with * *p* < 0.05, ** *p* < 0.01, *** *p* < 0.001, and **** *p* < 0.0001 versus control.

Consistent with the transcriptional results, protein expressions in Cal-27 and SCC25 cells treated with APW also altered in a similar trend. As shown in Figure 4A–D, the expressions of LRP6, DVL2, DVL3, Naked1, and Wnt5a/b proteins were significantly reduced after APW treatment in a concentration-dependent manner. Furthermore, expression of β-catenin, Met, and cyclin D1 proteins was also suppressed by APW treatment (Figure 4E–H). Their results further indicated that APW could suppress the Wnt/β-catenin signaling pathway in TSCC cells.

### 2.4. APW-Induced Apoptosis Associated with Wnt/β-Catenin Suppression and Mitochondrial Dysfunction

**Figure 5:**
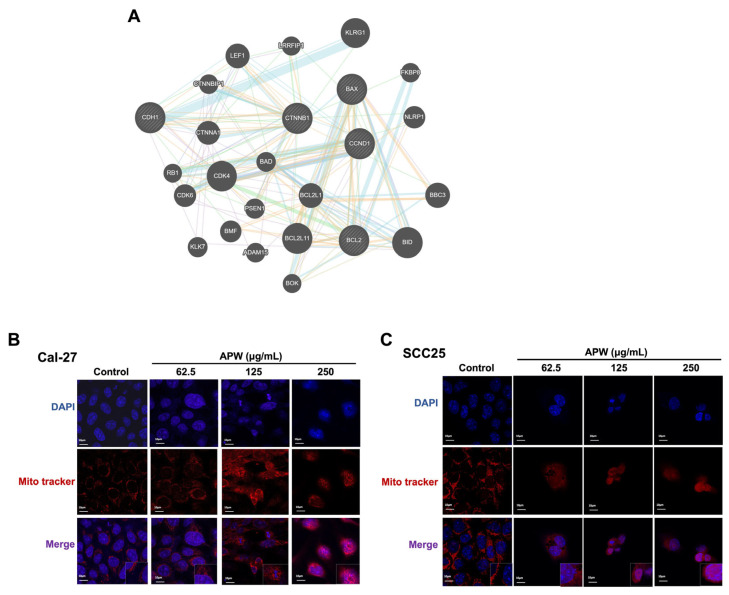
APW triggered mitochondrial dysfunction in Cal-27 and SCC25 cells. (**A**) STRING-based protein–protein interaction network showing the known/predicted associations between β-catenin and apoptosis-related proteins relevant to APW treatment. Confocal microscopy images showing mitochondrial morphology in (**B**) Cal-27 and (**C**) SCC25 cells treated with APW (62.5, 125, 250 μg/mL) for 48 h, stained with DAPI (nuclei, blue) and MitoTracker Red CMXRos (mitochondria, red). Scale bar = 10 μm.

**Figure 6:**
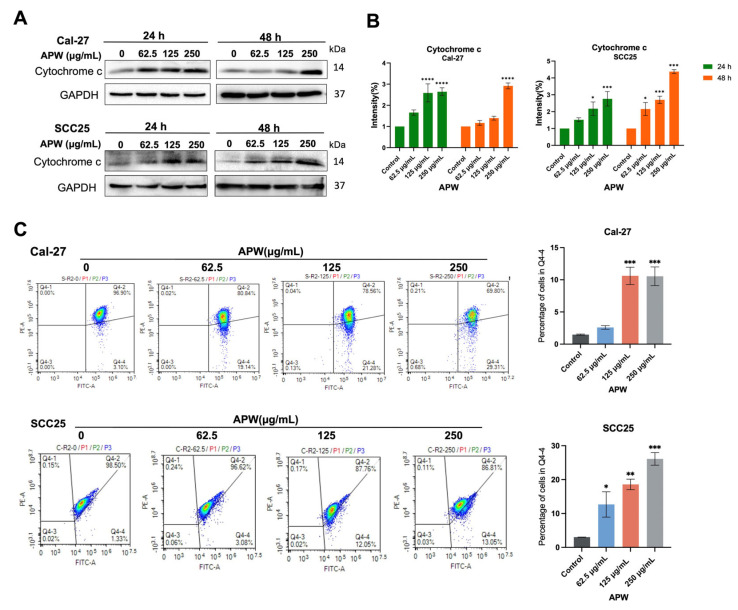
APW promoted cytochrome c release in Cal-27 and SCC25 cells. Cal-27 and SCC25 cells treated with APW (62.5, 125, and 250 μg/mL) for 24 and 48 h were subjected to Western blot analysis for cytosolic cytochrome c. (**A**) Representative blots of Cal-27 and SCC25 cells and (**B**) quantification of band intensities were shown. (**C**) Flow cytometry analysis of mitochondrial membrane potential (ΔΨm) of Cal-27 and SCC25 cells after APW treatment was performed using JC-1 staining. Representative JC-1 dot plots were shown on left panel. Percentage of cells with decreased mitochondrial membrane potential (in Q4-4) was shown on right panel. Data are presented as mean ± SD (*n* = 3). Statistical significance was determined using one-way ANOVA, with * *p* < 0.05, ** *p* < 0.01, *** *p* < 0.001, and **** *p* < 0.0001 versus control.

Based on our previous findings that APW inhibited Wnt/β-catenin signaling and induced apoptosis through BCL-2/BAX regulation and activation of caspases, as β-catenin was identified as a key APW-regulated molecule in our targeted analysis of Wnt pathway-related candidates, STRING analysis was performed to explore its interaction network with apoptosis-related proteins (Figure 5A and Table S4). Given that BAX and BCL-2 are the key regulators of mitochondrial-mediated apoptosis, the effects of APW on mitochondrial integrity were examined. By using MitoTracker staining, pronounced mitochondrial fragmentation was found in APW-treated Cal-27 and SCC25 cells, indicating structural disruption (Figure 5B,C). In addition, the levels of cytochrome c released into the cytosol of TSCC cells were increased after APW treatment in a concentration-dependent manner, which was in line with mitochondrial outer membrane permeabilization (Figure 6A,B). The results of the JC-1 assay further showed the loss of mitochondrial membrane potential after APW treatment, confirming mitochondrial dysfunction in Cal-27 and SCC25 cells (Figure 6C). Taken together, these findings suggested that APW-induced apoptosis is associated with both suppression of Wnt/β-catenin signaling and mitochondrial dysfunction, including altered BAX/BCL-2 balance, cytochrome c release, and loss of mitochondrial membrane potential.

### 2.5. APW Suppressed EMT and Migration in TSCC Cells

**Figure 7:**
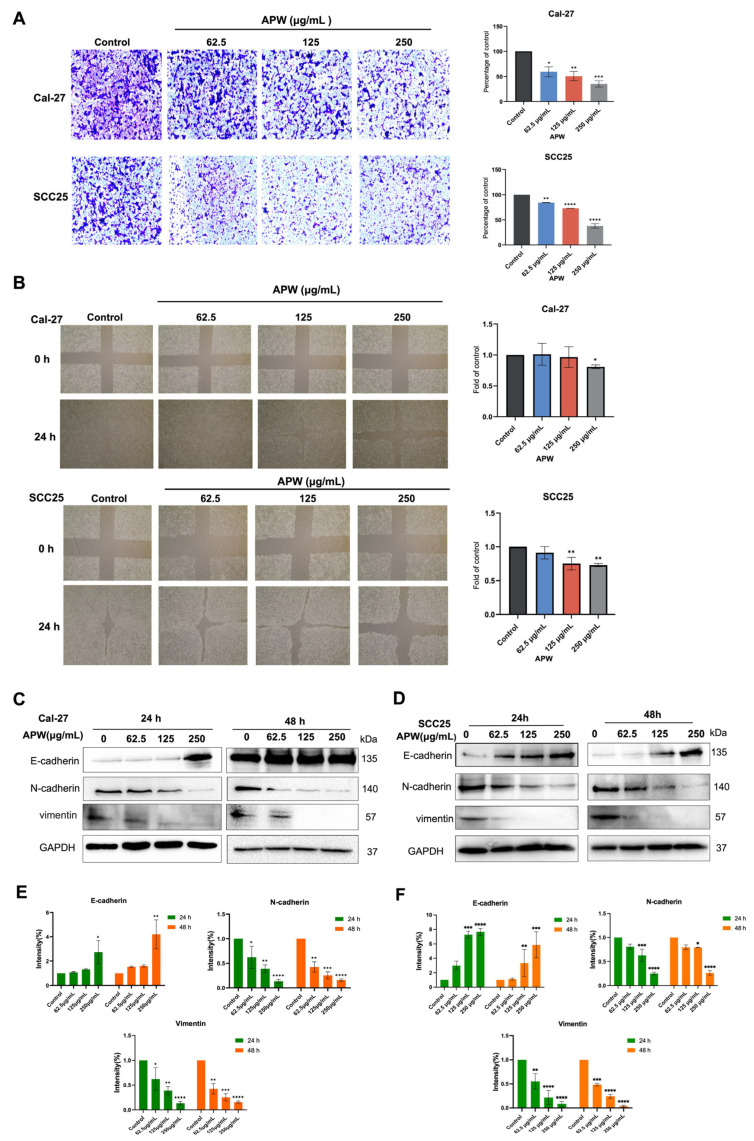
APW reduced migration and modulated EMT-related protein expressions in Cal-27 and SCC25 cells. (**A**) In transwell migration assay, Cal-27 and SCC25 cells were treated with increasing concentrations of APW (62.5, 125, and 250 μg/mL) for 24 h, and the number of migrated cells was quantified after crystal violet staining. Data are presented as mean ± SD (*n* = 3). (**B**) In wound healing assay, Cal-27 and SCC25 cells were treated with APW at indicated concentrations for 16 h, and wound area was photographed at 0 and 24 h. The wound closure area was calculated and normalized to control wells. Data are presented as mean fold of control ± SD (*n* = 3). Representative photographs of transwell migration and scratch wound healing assays were shown on the left panel (magnification 400×). The quantified data were shown on the right panel. Cal-27 and SCC25 cells were treated with APW (62.5, 125, 250 μg/mL) for 24 h or 48 h and subjected to Western blot analysis of EMT-related proteins. Representative blots was shown in (**C**,**D**). Quantification of band intensities were shown in (**E**,**F**). Statistical significance was determined using one-way ANOVA, with * *p* < 0.05, ** *p* < 0.01, *** *p* < 0.001, and **** *p* < 0.0001 versus control.

Apart from the findings of apoptosis induction by APW through Wnt/β-catenin inhibition and mitochondrial dysfunction, the effect of APW on EMT, a process related to cancer cell migration, has further been explored. In wound-healing and migration assays, in which the cells were treated with APW for 24 h, results showed that APW could markedly reduce the motility (Figure 7A) and migratory abilities (Figure 7B) of Cal-27 and SCC25 cells. The expressions of EMT-related markers in both cell lines were determined after 24 or 48 h APW treatment. Up-regulation of E-cadherin (epithelial marker) expression, accompanied by concomitant suppression of N-cadherin and vimentin (mesenchymal markers) expressions, was observed (Figure 7C–F). These findings indicated that APW not only promoted apoptotic cell death but also impeded tumor progression by inhibiting EMT-associated migration.

### 2.6. APW Inhibited the Growth of Tongue Cancer Xenografts in Mice

**Figure 8:**
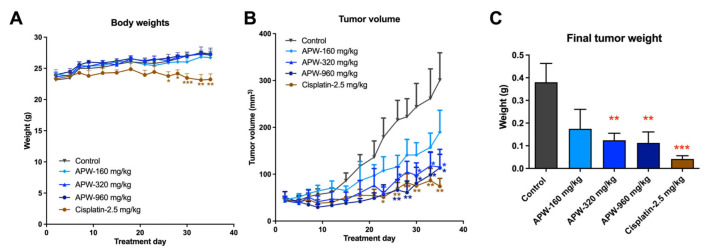

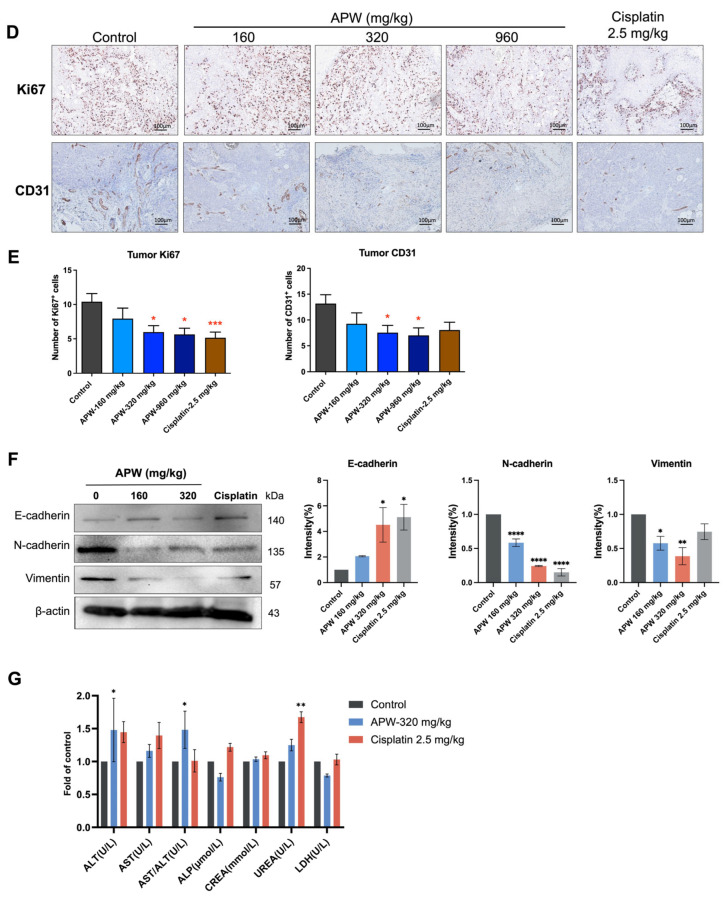
APW suppressed tumor growth, angiogenesis, and EMT in vivo. Cal-27 tumor-bearing mice were treated with APW (160, 320, or 960 mg/kg), cisplatin (2.5 mg/kg), or vehicle (Control) for 5 weeks. (**A**) Body weight changes of mice during the treatment period. (**B**) Tumor growth curves and (**C**) final tumor weights of mice in different treatment groups. (**D**) Tumors of mice from different treatment groups were subjected to IHC staining with Ki67 and CD31 antibodies. Representative immunohistochemical images of Ki67- and CD31-stained tumor sections. Scale bars = 100 μm. (**E**) Quantification of Ki67- and CD31-positive cells in tumor sections. (**F**) Tumor tissues of APW (160 and 320 mg/kg) or cisplatin (2.5 mg/kg)-treated mice were subjected to Western blot analysis of EMT-related proteins (E-cadherin, N-cadherin, and vimentin). Representative blots were on the left panel, and quantification of band intensities was shown on the right panel. (**G**) Bar chart showing the plasma levels of ALT, AST, ALP, CREA, UREA, and LDH in plasma collected from mice treated with APW (320 mg/kg) or cisplatin (2.5 mg/kg). Data were presented as mean ± SEM. Number of mice: control = 13; APW-160 mg/kg = 12; APW-320 mg/kg = 13; APW-960 mg/kg = 14; cisplatin-2.5 mg/kg = 13. Statistical significance was determined using one-way ANOVA, with * *p* < 0.05, ** *p* < 0.01, *** *p* < 0.001, and **** *p* < 0.0001 versus control.

To validate the in vivo anti-tumor efficacy of APW, a tongue-cancer Cal-27 xenograft mouse model was used. As shown in Figure 8A, the body weights of mice have not been affected by 5-week APW treatments; however, the body weight of mice treated with cisplatin was decreased during treatment, with significant decreases observed from 26 days of treatment. Meanwhile, the tumor volume of APW-treated mice was found to be significantly reduced after 26 days of treatments (*p* < 0.01) when compared with control untreated mice (Figure 8B). APW treatments at 320 and 960 mg/kg significantly suppressed tumor growth, and the final tumor weights were reduced (*p* < 0.01, Figure 8C). The tumors were subjected to immunohistochemical staining; results showed that the expressions of the proliferation marker Ki67 and the angiogenesis marker CD31 were markedly reduced in tumors of APW-treated mice (Figure 8D,E). To further assess whether APW inhibits EMT in vivo, western blot analysis of proteins in tumor tissues was performed. Consistent with the in vitro findings, there was increased E-cadherin and decreased N-cadherin & vimentin expression levels in APW-treated mice tumors (Figure 8F). These results indicate that APW suppressed EMT in vivo, thereby contributing to its inhibitory effects on tumor metastasis. On the other hand, biochemical analysis of plasma was performed. Results demonstrated that APW treatment (320 mg/kg) did not cause significant hepatic or renal toxicity, as reflected by the relatively stable levels of ALT, AST, ALP, CREA, and UREA compared with untreated control and cisplatin-treated mice (Figure 8G).

## 3. Discussion

In the present study, APW exerted potent anti-tumor effects against tongue squamous cell carcinoma both in vitro and in vivo. Our findings revealed that APW could significantly suppress the proliferation and migration of Cal-27 and SCC25 cells and induce apoptosis in a concentration-dependent manner. Mechanistic investigation showed that APW triggered apoptosis through dual pathways—suppression of the Wnt/β-catenin signaling cascade and activation of mitochondrial-mediated cell death—thereby exerting a multifaceted anti-tumor effect. Furthermore, APW effectively reversed EMT and inhibited angiogenesis in vivo.

The AP water extract used in the present study contains diterpernoids andrographolide, neoandrographolide, deoxyandrographolide, dehydroandrographolide, and other components. Previous studies have reported the anti-tumor effect of individual components, e.g., andrographolide [25] and dehydroandrographolide [26], in oral cancer. Nonetheless, the water extract of AP, which is the dosage form commonly consumed by patients, has not yet been proven to be efficacious in TSCC animal models. Here, we demonstrated for the first time that AP water extract could suppress the tumor growth in CAL-27 xenograft-bearing mice. The effective dose in this model was found to be 320 mg/kg, which is equivalent to the human clinical dose recommended in the Chinese Pharmacopoeia. It is anticipated that the potent anti-tumor effect of AP water extract would be the combined effects of multiple components.

The balance between pro-apoptotic and anti-apoptotic members of the BCL-2 family plays a pivotal role in the regulation of mitochondrial integrity and apoptosis [27,28]. Our data demonstrated that APW treatment in TSCC cells upregulated BAX but downregulated BCL-2 expression, thereby disrupting mitochondrial homeostasis and activating the caspase cascade. This observation aligns with other studies in which natural compounds could induce intrinsic apoptosis through the regulation of mitochondrial membrane permeability and cytochrome c release [29,30]. Moreover, the observed increases in cleaved apoptotic proteins confirmed that APW activated downstream caspase-dependent cell death signaling.

In addition to mitochondrial dysfunction, our results revealed that APW markedly suppressed the Wnt/β-catenin signaling pathway. APW treatment downregulated several upstream regulators of Wnt signaling—including LRP6, DVL2/3, NKD2, and Wnt5a/b—as well as β-catenin target genes such as CTNNB1, MYC, and CCND1. This inhibition resulted in decreased expression of β-catenin and Cyclin D1 at the protein level, suggesting that APW effectively blocks nuclear accumulation of β-catenin as well as transcriptional activation of oncogenic targets. Given the known crosstalk between Wnt/β-catenin signaling and mitochondrial apoptotic regulation [22,31], APW was found to be accompanied by both suppression of Wnt/β-catenin signaling and activation of mitochondrial apoptotic events. Nevertheless, our data has not yet illustrated the inhibition of Wnt/β-catenin signaling is a direct [32] or required mediator of APW-induced apoptosis, which was a limitation of the present study. Further studies using rescue or pathway perturbation approaches to determine whether Wnt/β-catenin inhibition is a direct mediator of APW-induced apoptosis will be performed.

EMT is an important biological process that contributes to cancer metastasis by allowing epithelial cells to obtain mesenchymal properties and migratory capability [33,34]. We observed that APW not only inhibited cell migration in vitro but also reversed EMT, as the increased E-cadherin and decreased N-cadherin and vimentin expressions in TSCC cells and tumors showed. These molecular changes indicate that APW maintained epithelial characteristics while suppressing the invasive ability of tongue cancer cells. Notably, the in vivo experiments further corroborated these findings, i.e., APW treatment inhibited tumor growth and angiogenesis and induced similar alterations in EMT-related markers in tumors. The hypothesis of the cancer pathological ecosystem was proposed recently, in which EMT was suggested to be conceptualized as a manifestation of cancer cells competition and adaptive response to adverse external microenvironmental stressors [35]. It is possible that APW may play a role in the balance of such a pathological esosystem in TSCC, which is worth further investigation.

Importantly, plasma biochemical analyses indicated that APW treatment at 320 mg/kg did not cause significant hepatotoxicity or nephrotoxicity, suggesting favorable biocompatibility and systemic safety. Such biochemical analyses have not been performed in the APW 960 mg/kg treatment group because the anti-tumor efficacy of APW at 320 and 960 mg/kg was similar; we focused on the expressions of EMT-related proteins and the safety of the “minimum effective dose” of APW, i.e., 320 mg/kg. Nevertheless, the anti-tumor efficacy of APW at 320 mg/kg was comparable to that of cisplatin in terms of reducing Ki67^+^ proliferative cells and CD31^+^ endothelial cells in tumors, yet with fewer adverse effects, underscoring its promise as a potential therapeutic candidate for tongue cancer.

## 4. Materials and Methods

### 4.1. Materials

Dried herb of AP was purchased from a renowned herbal retailer in Hong Kong and was authenticated by botanist Dr. David Tai-Wai Lau, Curator of Shiu-Ying Hu Herbarium, School of Life Sciences, The Chinese University of Hong Kong. The voucher specimen with the number 3738 was deposited in the museum of the Institute of Chinese Medicine, The Chinese University of Hong Kong.

Human tongue squamous cell carcinoma (TSCC) Cal-27 and SCC25 cells were purchased from American Type Culture Collection (Manassas, VA, USA). RPMI1640 medium with 10% (*v*/*v*) fetal bovine serum (FBS) and 1% penicillin/streptomycin was used for culturing both cell lines. The cell culture media and supplements were purchased from Thermo Fisher Scientific (Waltham, MA, USA). The cells were maintained in an incubator at 37 °C with a humidified atmosphere with 5% CO_2_.

Male nude mice (aged 6–8 weeks) were supplied by the Laboratory Animal Services Center of the Chinese University of Hong Kong. The experimental procedures of the animal study were approved by the Animal Experimentation Ethics Committee of The Chinese University of Hong Kong (Ref. No. 21-287-MIS; approval date: 30 November 2021).

### 4.2. Chemical Analysis of AP Herb

The Agilent 1290 UHPLC with a 6530 QTOF system (Santa Clara, CA, USA) was used to conduct the analysis. The Agilent ZORBAX Eclipse Plus C18 RRHD column (2.1 × 150 mm, 1.8 µm) (Santa Clara, CA, USA), accompanied by a guard column (Agilent ZORBAX Eclipse Plus C18 UHPLC Guard, 2.1 × 5 mm, 1.8 µm) (Santa Clara, CA, USA) was used. The separation was conducted at 40 °C under gradient conditions at a flow rate of 0.5 mL/min. The liquid chromatographic profile was: Mobile phase: (A) 0.1% formic acid in deionized water and (B) 0.1% formic acid in acetonitrile; Gradient: 0–2 min, 5% B; 2–17 min, 5–40% B; 17–18 min, 40–44% B; 18–21 min, 44% B; 21–25 min, 44–54% B; 25–27 min, 54–100% B. The column was flushed with 100% B for 3 min and re-equilibrated for another 3 min after each injection. High-purity nitrogen was used as the curtain and collision gas with a flow rate of 10 L/min. The drying gas temperature was set at 350 °C, and the nebulizer pressure was set at 50 psi. Spectra were recorded in positive ion mode at a spray voltage of 4000 V. The mass scan range was between 100 and 1100 *m*/*z*. Agilent MassHunter Workstation Qualitative Analysis Software (Santa Clara, CA, USA, version B.07.00) was applied to perform the data analysis. Molecular Features Extraction was used to identify the chemical markers, andrographolide, neoandrographolide, deoxyandrographolide, and dehydroandrographolide, in the extract, and the result was compared with the *Andrographis paniculata* database established by Agilent MassHunter PCDL Manager Software (version B.07.00). The UPLC chromatogram of AP herb with chemical markers labeled was shown in Supplementary Information Figure S1, and the content of such chemical markers was listed in Table S1.

### 4.3. Preparation of AP Water Extract

Dried herbs of AP (500 g) were soaked in distilled water (5 L) for 1 h, followed by boiling in water with reflux for 1 h and then repeated once. The combined water extracts were filtered and concentrated under vacuum, followed by freeze-drying in a freeze-dryer (Labconoco, Kansas City, MO, USA) and finally became powder form. The AP water extract (APW) powder was stored in desiccator at room temperature. The extraction yield of APW was 17.4% (*w*/*w*).

### 4.4. Cytotoxicity Assay

Human TSCC Cal-27 and SCC25 cells (5 × 10^3^ cells per well) were seeded in 96-well culture plates and incubated overnight. Then, APW at various concentrations was added into the wells with adhered cells. After 48 h of incubation, the cells in the plates were subjected to a colorimetric MTT assay for cell viability assessment.

### 4.5. Colony Formation Assay

Cal-27 and SCC25 cells were seeded in 6-well plates (1 × 10^5^ cells per well). After 12 h of incubation, cells were treated with different concentrations of APW and further incubated for 48 h. Cells were then washed once with phosphate buffered saline (PBS) and fixed with 4% paraformaldehyde in PBS. Subsequently, cells were stained with 0.1% crystal violet solution (1 mL per well) for 20 min and rinsed thoroughly with water. The plates with stained cells were air-dried at room temperature, and representative images of each well were taken.

### 4.6. Annexin V/7-AAD Apoptosis Assay

Cal-27 and SCC25 cells were seeded in 6-well plates overnight, and then treated with APW at concentrations of 62.5, 125, 250, and 500 µg/mL. After 48 h of incubation, PE-Annexin V and 7-AAD staining were conducted in these cells according to the manufacturer’s instructions (TransGen, Beijing, China). Flow cytometric analysis of the stained cells was performed using a NovoCyte Advanteon BVYG flow cytometer (Agilent, Santa Clara, CA, USA). Data compensation and quadrant gating were performed with FlowJo software (version 10.8.1). Cells in quadrant Q2 (Annexin V^+^/7-AAD^+^) were defined as late apoptotic, those in Q3 (Annexin V^+^/7-AAD^−^) as early apoptotic, Q1 (Annexin V^−^/7-AAD^+^) as necrotic, and Q4 (Annexin V^−^/7-AAD^−^) as viable. The apoptotic rate was calculated as the sum of early and late apoptotic cells (Q2 + Q3), normalized to the corresponding vehicle control.

### 4.7. Western Blot Analysis

Cal-27 and SCC25 cells seeded in 6-well plates were treated with various concentrations of APW for 24 or 48 h. After treatment, cells were collected, washed with PBS, and lysed using RIPA buffer (Thermo Fisher Scientific). BCA assay was used to determine the protein concentrations. Next, whole-cell proteins were separated on 10–12.5% SDS-polyacrylamide gels and transferred onto PVDF membranes (0.45 or 0.22 µm, Immobilon, Millipore, Burlington, MA, USA). The membranes were blocked with 5% fat-free milk for 2 h and then incubated with primary antibodies overnight at 4 °C with shaking. Detailed information on antibodies is listed in Supplementary Information Table S2. The following day, after three 10-min washes with TBST, membranes were incubated with HRP-conjugated secondary antibodies (rabbit or mouse) for 1 h. Protein bands were detected using a ChemiDoc XRS+ molecular imager (Bio-Rad Laboratories, Hercules, CA, USA).

### 4.8. Mitochondrial Morphology Analysis

Cal-27 and SCC25 cells were cultured to 70–80% confluence and then stained with MitoTracker^®^ probes (Thermo Fisher Scientific, Waltham, MA, USA) at a working concentration of 25–500 nM for 15–45 min in serum-free medium. Then, the staining solution was replaced with fresh medium, and the stained cells were observed using a Live-SR Super-resolution/TIRF microscope (Nikon, Tokyo, Japan).

### 4.9. JC-1 Probe Detection

Cal-27 and SCC25 cells were seeded in 6-well plates overnight. After APW treatment, cells were collected and washed twice with pre-cooled PBS to remove culture medium and residual debris. The cells were then incubated with JC-1 dye at a concentration of 2–10 μM under dark conditions at 37 °C for 20–30 min to allow sufficient accumulation in the mitochondria. After incubation, cells were washed 1–2 times with pre-warmed PBS to remove unbound dye and then resuspended in PBS for flow cytometry analysis. The JC-1 fluorescence was measured using Cell Analyzer Agilent NovoCyte Advanteon BVR (Agilent, Santa Clara, CA, USA), recording both green (~529 nm, JC-1 monomers) and red (~590 nm, JC-1 aggregates) signals. The red/green fluorescence ratio was calculated using FlowJo software. The experimental steps were referenced to the MitoProbe™ JC-1 Assay Kit (Thermo Fisher Scientific).

### 4.10. Transwell Migration Assay

Cal-27 and SCC25 cells were treated with increasing concentrations of APW (0, 62.5, 125, and 250 μg/mL) for 24 h. Then, the harvested cells were seeded in the upper chamber of Transwell (pore size 8 μm, Corning, Union City, CA, USA) with serum-free medium, while the lower chamber contained complete medium with 10% FBS. After 24 h incubation, non-migrated cells in the upper chamber were removed, and cells on the lower surface of the membrane were fixed and stained with crystal violet. The stained cells were counted in three random fields while the migration rate was expressed as the average cell count per field.

### 4.11. Wound Healing Assay

CAL-27 and SCC25 cells (2 × 10^5^ in 1 mL) were seeded in 6-well plates and incubated overnight. By using a sterile 200 μL pipette tip, a scratch was made across the cell monolayer, and detached cells were removed by gently washing with PBS. Immediately after scratching (0 h), images of the wound area were captured using the EVOS^TM^ XL Core Imaging System (Thermo Fisher Scientific, Waltham, MA, USA). The cells were then incubated in serum-free medium to minimize proliferation for 24 h. Subsequently, the cells in the control well were maintained in normal medium, while the cells in the treatment wells were incubated with different concentrations of APW (0, 62.5, 125, and 250 μg/mL) for an additional 24 h. Migration was quantified by measuring the wound area and calculating the percentage of wound closure using ImageJ software (version 2.3.0/1, Bethesda, MD, USA).

### 4.12. RNA Extraction, PCR, Real-Time Quantitative PCR (RT-qPCR)

Cal-27 and SCC25 cells were treated with APW at 0, 62.5, 125, and 250 μg/mL for 24 or 48 h and harvested for RNA extraction. On the other hand, tumor tissues were collected from Cal-27 xenograft-bearing mice after APW treatment. Total RNA was extracted from TSCC cells and tumor tissues using TRIzol reagent (Invitrogen). One microgram of RNA was then reverse transcribed using the EasyScript^®^ One-Step SuperMix (TransGen, Beijing, China). The SYBR Green PCR reagent kit (Invitrogen) was used for quantitative real-time PCR (RT-qPCR) on the LightCycler^®^ 480 Instrument II (Roche, Basel, Switzerland). The gene expression values were normalized to GAPDH. Primers were synthesized by IGE Biotechnology LTD (Guangzhou, China). Primer sequences are shown in Supplementary Information Table S3.

### 4.13. Human Tongue Cancer Xenograft Mouse Model

Male nude mice aged 6–8 weeks were inoculated subcutaneously with Cal-27 cells (5 × 10^6^ per mouse) on the flank. Treatments of APW or cisplatin were started 14 days after cell inoculation. Dosages of APW for animal studies were calculated in accordance with the Chinese Pharmacopeia 2020, in which the recommended dosages of AP are 6–9 g. Taken 9 g, for example, the extraction yield of APW was 17.4%; the human dosage of APW would be 26.1 mg/kg. By using the conversion factors in US FDA guidance “Conversion of Animal Doses to Human Equivalent Doses” [36], APW dosage for mice would be around 320 mg/kg. Therefore, half (160 mg/kg) and three times (960 mg/kg) of such dosages have been used. All mice (total of 70 mice) bearing tumors were included during the experiment, and tumor sizes were measured before treatments. Mice with similar tumor sizes were pooled and then randomly assigned to 5 treatment groups: control, APW-160 mg/kg, APW-320 mg/kg, APW-960 mg/kg, and cisplatin-2.5 mg/kg treatment groups. They were orally administered APW in 2 mL daily for 5 weeks, while cisplatin in 0.1 mL was given intraperitoneally to mice every 3 days for 5 weeks. Mice in the control group were orally administered 2 mL of distilled water. Tumor sizes were measured using a caliper three times a week by a second investigator who was unaware of the treatments. Tumor volume was calculated using the formula [d^2^ × D/2, where d and D are the shortest and longest diameters in mm, respectively]. The tumor size of a few mice exceeded the maximum humane endpoint, so that the mice were euthanized before the end of the experiment. Thus, the data points have not been included in the analysis. At the end of the experiment, mice were anesthetized and whole blood was collected. Then the mice were euthanized by cervical dislocation, and tumors were collected for further analysis. Tumor weights were recorded. Levels of alanine aminotransferase (ALT), aspartate aminotransferase (AST), alkaline phosphatase (ALP), creatinine (CREA), urea (UREA), and lactate dehydrogenase (LDH) in plasma were determined using a Clinical Chemistry Analyzer (BS-230, Mindray, Shenzhen, China).

### 4.14. Immunohistochemistry (IHC) Staining

Mouse tumor tissue sections were processed following standard immunohistochemistry protocols. Sections were deparaffinized in xylene, rehydrated through graded series of ethanol, and then subjected to heat-induced epitope retrieval in 10 mM sodium citrate buffer (pH 6.0) for 15 min in a microwave. Endogenous peroxidase activity was quenched with 3% hydrogen peroxide for 10 min at room temperature. After blocking with 5% bovine serum albumin (BSA) for 2 h, primary antibodies (CD31, 1:20; Ki67, 1:150) diluted in BSA were added to the sections, which were incubated overnight at 4 °C. After washing, sections were incubated with a biotinylated secondary antibody for 1.5 h at room temperature, developed using 3,3-diaminobenzidine tetrahydrochloride (DAB). Staining was visualized and imaged under a bright-field microscope (BX43 Manual System, Olympus, Tokyo, Japan).

### 4.15. Statistical Analysis

In vitro data were expressed as mean ± standard deviation (SD), and in vivo data were expressed as mean ± standard error of the mean (SEM). All data were analyzed for normal distribution and homogeneity of variance. One-way analysis of variance (ANOVA) was applied for means comparison using GraphPad PRISM software (version 10.0, GraphPad Software, Boston, MA, USA). A *p*-value less than 0.05 was considered statistically significant.

## 5. Conclusions

**Figure 9:**
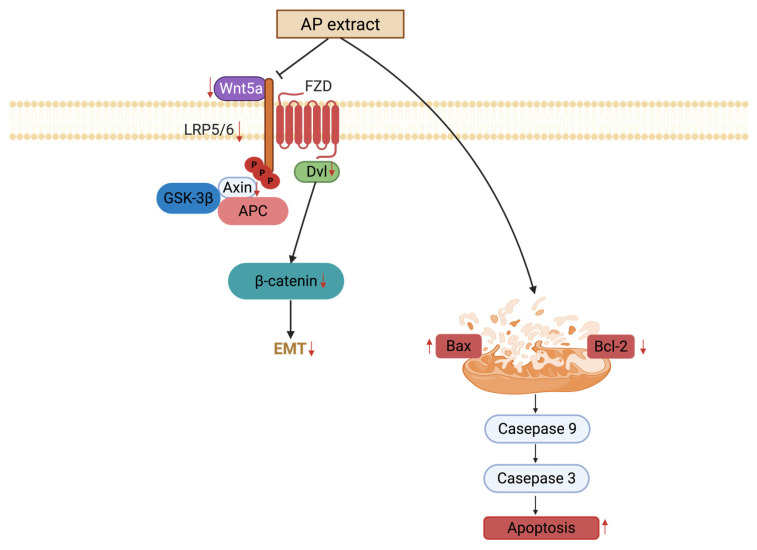
Schematic illustration of the proposed effects of *Andrographis paniculata* water extract on tongue squamous cell carcinoma. Arrows (↑) indicate upregulation, arrows (↓) indicate downregulation. Created in BioRender. Huang, J. (2026) https://BioRender.com/c6llesb (accessed on 17 April 2026).

Our findings demonstrate that APW exerted anti-tumor effects through multiple interconnected mechanisms: inhibition of the Wnt/β-catenin pathway, induction of mitochondrial-mediated apoptosis, and suppression of EMT-related protein expressions (Figure 9). By targeting tumor growth, APW can be regarded as a promising herbal medicine for further clinical translation in tongue squamous cell carcinoma management.

## Data Availability

The original contributions presented in this study are included in the article/Supplementary Materials. Further inquiries can be directed to the corresponding authors.

## Supplementary Materials

The following supporting information can be downloaded at: https://www.mdpi.com/article/10.3390/ijms27093772/s1.
