# Supplementary material for: Andrographis paniculata Inhibits Tongue Squamous Cell Carcinoma via Regulating Wnt/β-Catenin Signaling and Epithelial-Mesenchymal Transition
Source: Int J Mol Sci. 2026 Apr 23;27(9):3772. doi: 10.3390/ijms27093772 (PMC13164503; doi:10.3390/ijms27093772)
Supplement: Supplementary file 1 [file ijms-27-03772-s001.zip › ijms-4253083-supplementary.pdf]

# *Andrographis paniculata* Inhibits Tongue Squamous Cell Carcinoma via Regulating Wnt/β-Catenin Signaling and Epithelial-Mesenchymal Transition

**Grace Gar-Lee Yue [1,†], Jingyi Huang [1,†], Xiaotong Lu [1], Julia Kin-Ming Lee [2], Si Gao [2], Jason Ying Kuen Chan [3,*] and Clara Bik-San Lau [1,4,*]**

[1] Department of Pharmacology and Pharmacy, Li Ka Shing Faculty of Medicine, The University of Hong Kong, Pokfulam, Hong Kong SAR, China
[2] Institute of Chinese Medicine, The Chinese University of Hong Kong, Shatin, Hong Kong SAR, China
[3] Department of Otorhinolaryngology, Head and Neck Surgery, The Chinese University of Hong Kong, Shatin, Hong Kong SAR, China
[4] School of Chinese Medicine, Li Ka Shing Faculty of Medicine, The University of Hong Kong, Pokfulam, Hong Kong SAR, China
* Correspondence: jasonchan@ent.cuhk.edu.hk (J.Y.K.C.); cbslau@hku.hk (C.B.-S.L.)
† These authors contributed equally to this work.

## Supplementary Information

**Table S1. The content of chemical markers in herbal material of *Andrographis paniculata***

| Chemical markers | Content % (w/w of raw herb) |
|---|---|
| Andrographolide | 1.176 ± 0.085 |
| Neoandrographolide | 0.365 ± 0.027 |
| Deoxyandrographolide | 0.306 ± 0.028 |
| Dehydroandrographolide | 0.579 ± 0.049 |

**Table S2. Antibodies used in this study.**

| Protein names | Company | Cat. No. |
|---|---|---|
| LRP6 | Cell signaling technology | 3395T |
| Dvl3 | Cell signaling technology | 3218T |
| Dvl2 | Cell signaling technology | 3224T |
| Naked1 | Cell signaling technology | 2262T |
| Wnt5a/b | Cell signaling technology | 2530T |
| Met | Cell signaling technology | 8198T |
| CCND1 | Cell signaling technology | 2978T |
| β-catenin | Cell signaling technology | #4967 |
| Cytochrome c | Santa cruz biotechnology | 11940S |
| vimentin | Cell signaling technology | 5741T |
| N-cadherin | Cell signaling technology | 13116S |
| E-cadherin | Cell signaling technology | 3195S |
| BCL-2 | Cell signaling technology | 3498S |
| BAX | Cell signaling technology | 41162S |
| Cleaved Caspase-3 | Cell signaling technology | 9664T |
| Cleaved Caspase-9 | Cell signaling technology | 52873T |
| Cleaved PARP | Cell signaling technology | 5625T |
| Cleaved Caspase-7 | Cell signaling technology | 8438T |
| Caspase-3 | Cell signaling technology | 14220T |
| PARP | Cell signaling technology | 9542T |
| Caspase-7 | Cell signaling technology | 12827T |
| Caspase-9 | Cell signaling technology | 9508T |
| GAPDH | Cell signaling technology | 2118S |
| β-actin | Abclonal | MA5-15739 |
| CD31 | Dianova | DIA-310 |
| Ki67 | Abcam | ab16667 |

**Table S3. Sequences of qRT-PCR primers used in this study**.

| Gene | Forward (5'-3') | Reverse (5'-3') |
|---|---|---|
| LRP6 | CCCATTTGTGTTTGATGTCTCC | AATGCGTCTCAAGTCTGTCC |
| AXIN1 | CCATACAGGATCCGTAAGCAG | GAACTTCTGAGGCTCCACG |
| WNT5A | TCGCCCAGGTTGTAATTGAAG | TGAGAAAGTCCTGCCAGTTG |
| DVL2 | AAGTCTATGGATCAGGATTTCGG | GGATTATCTGAGGACACCAGC |
| DVL3 | CCTCCATCACCAGTTCCATC | GTAATCTTGAGCCACATGCG |
| NKD2 | ACAGGAGGTTGTCTGCACACG | GACTTGAGGAACTGCTTCTCCG |
| CD44 | TCTTCAACCCAATCTCACACC | TCCTGTCCAAATCTTCCACC |
| CCND1 | CATCTACACCGACAACTCCATC | TCTGGCATTTTGGAGAGGAAG |
| CTNNB1 | GTTCAGTTGCTTGTTCGTGC | GTTGTGAACATCCCGAGCTAG |
| JUN | AGCCCAAACTAACCTCACG | TGCTCTGTTTCAGGATCTTGG |
| LEF1 | GTCAACTCCAAACAAGGCATG | CGTGATGGGATATACAGGCTG |
| MET | GACTCCTACAACCCGAATACTG | ATAGTGCTCCCCAATGAAAGTAG |
| MMP7 | TTCCAAAGTGGTCACCTACAG | AGTTCCCCATACAACTTTCCTG |
| MYC | TTCGGGTAGTGGAAAACCAG | AGTAGAAATACGGCTGCACC |
| TCF7/1 | GTCTACTCCGCCTTCAATCTG | GTGGGCTGTTGAAATGTTCG |
| BAX | GACATGTTTTCTGACGGCAAC | AAGTCCAATGTCCAGCCC |
| BCL-2 | GTGGATGACTGAGTACCTGAAC | GCCAGGAGAAATCAAACAGAGG |
| CASP3 | ACTGGACTGTGGCATTGAG | GAGCCATCCTTTGAATTTCGC |
| CASP9 | CCAACCCTAGAAAACCTTACCC | TCTGCATTTCCCCTCAAACTC |
| GAPDH | GAACGGGAAGCTCACTGG | GCCTGCTTCACCACCTTCT |

**Table S4. STRING settings of candidate genes/proteins used in this study**

| Gene | Category | Assay |
|---|---|---|
| WNT5A | ligand | qPCR/WB |
| LRP6 | receptor | qPCR/WB |
| DVL2 | adaptor | qPCR/WB |
| DVL3 | adaptor | qPCR/WB |
| NKD2 | regulator | qPCR |
| CTNNB1 | central mediator | qPCR/WB |
| CCND1 | downstream | qPCR/WB |
| MYC | downstream | qPCR |
| JUN | downstream | qPCR |
| MET | downstream | qPCR/WB |

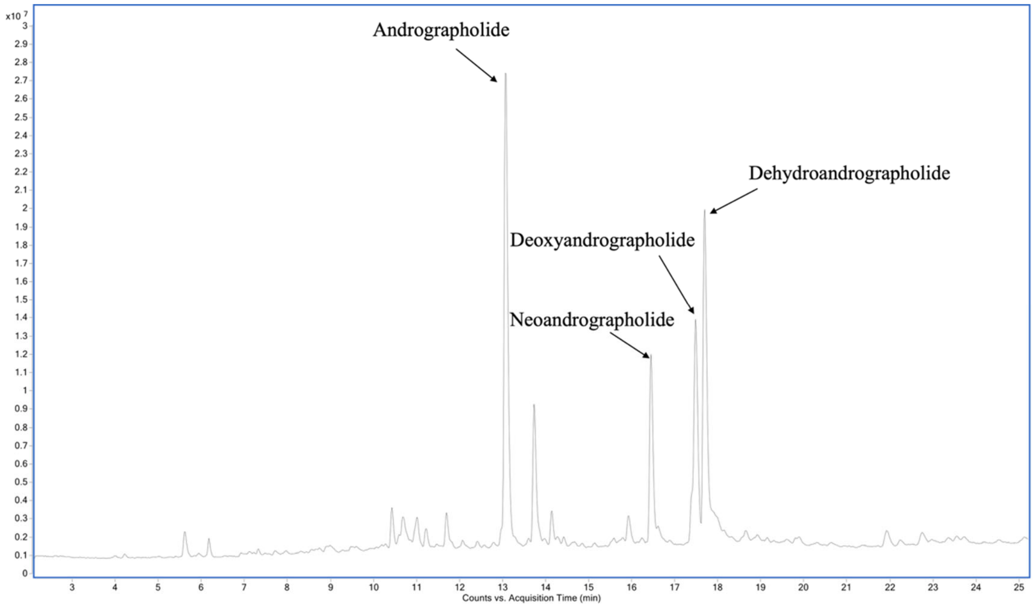


**Figure S1. Total ion chromatogram of *Andrographis paniculata* herb.**

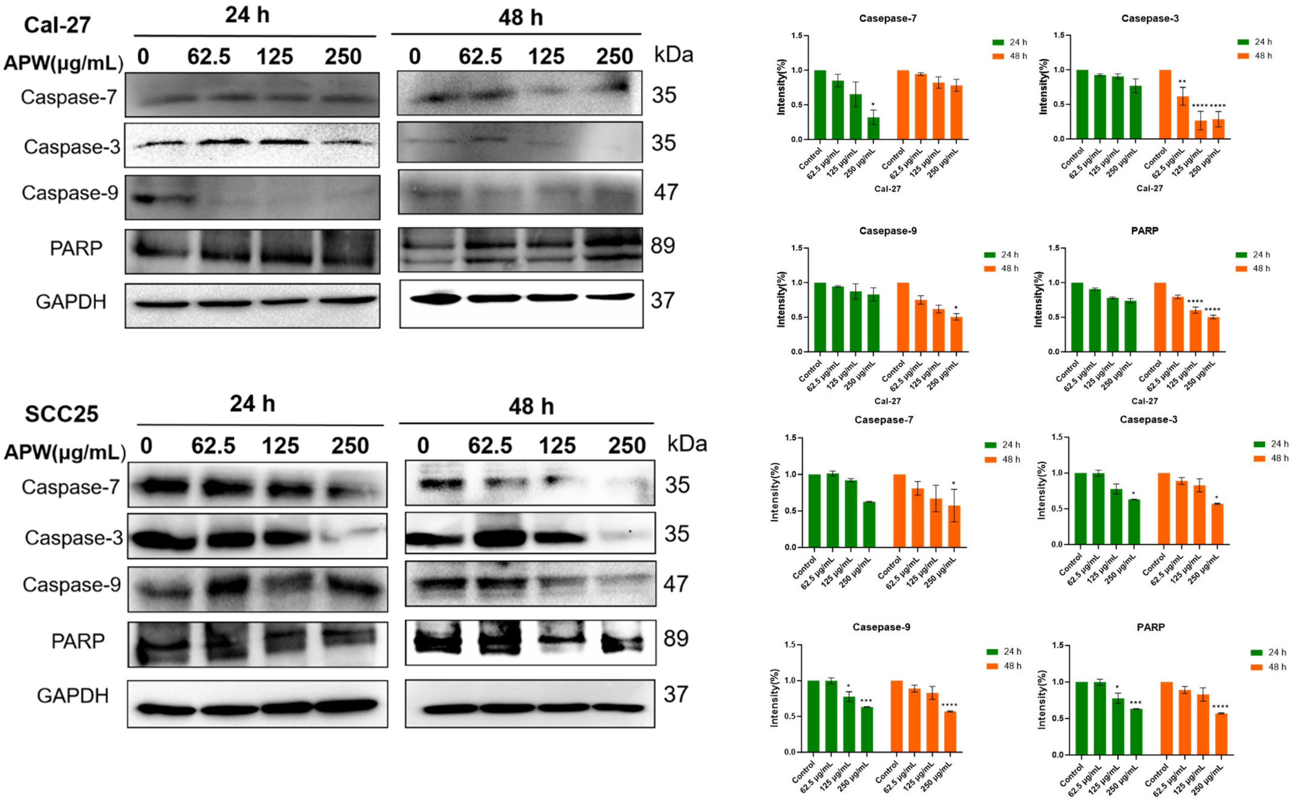


**Figure S2. Western blot analysis of apoptosis-related proteins following APW treatment in tongue cancer cells.**

Cal-27 and SCC25 cells were treated with increasing concentrations of APW (0, 62.5, 125, and 250 μg/mL) for 24 h or 48 h. Protein expression levels of apoptosis-associated markers. Densitometric quantification of Western blot bands was performed using ImageJ and normalized to GAPDH. Data are presented as mean ± SD of three independent experiments. Statistical significance was determined using one way ANOVA (*$p < 0.05$, **$p < 0.01$, ***$p < 0.001$).
